# Early childhood growth patterns and malnutrition in rural Southern India: a longitudinal study of a birth cohort

**DOI:** 10.3389/fpubh.2026.1783540

**Published:** 2026-05-14

**Authors:** Kalpana Betha, Shailendra Dandge, Govind Kusneniwar, Sameer Valsangkar, Sirshendu Chaudhuri, Varun Agiwal, Nirupama A. Y, Hira Pant, G. V. S. Murthy, P. S. Reddy

**Affiliations:** 1 Department of Obstetrics and Gynecology, MediCiti Institute of Medical Sciences, Hyderabad, Telangana, India; 2 Society for Health Allied Research and Education India (SHARE INDIA), Hyderabad, Telangana, India; 3 Department of Pharmacology, MediCiti Institute of Medical Sciences, Hyderabad, Telangana, India; 4 All India Institute of Medical Sciences, Bibinagar, Hyderabad, Telangana, India; 5 Department of Community Medicine, MediCiti Institute of Medical Sciences, Hyderabad, Telangana, India; 6 Department of Medicine, Indian Institute of Public Health Hyderabad, Hyderabad, Telangana, India; 7 Pragyaan Sustainable Health Outcomes (PRASHO), Hyderabad, Telangana, India; 8 School of Medicine, University of Pittsburgh, University of Pittsburgh, Pittsburgh, PA, United States

**Keywords:** India, malnutrition, preschool children, stunting, wasting

## Abstract

**Background:**

Malnutrition remains a pervasive problem among Indian children, but cross-sectional surveillance surveys and studies fail to identify secular trends and growth patterns. The study analyzed longitudinal data from the Longitudinal Indian Family hEalth (LIFE) birth cohort, established in 2009 to examine early childhood development. The field area for the LIFE cohort consists of rural to peri-urban villages in Medchal-Malkajgiri district, Telangana State, India, located approximately 40 km from Hyderabad, Telangana, India. This setting features predominantly agrarian households with socioeconomic challenges. Growth and malnurtition patterns in normal-birth-weight (NBW) and low-birth-weight (LBW) children were compared at multiple time points.

**Methods:**

Five hundred and forty children were included. Trained staff recorded height/weight via standardized methods every six months (± two months) until 24 months, then yearly until 60 months. Anthropometric indices were stunting (HAZ < −2 SD), wasting (WHZ < −2 SD), and underweight (WAZ < −2 SD). Group differences were analyzed using proportion tests and unpaired *t*-tests to compare growth patterns between LBW and NBW, and to assess sex-based differences between boys and girls, following normality checks. Survival curves depicted trends and LBW vs. NBW differences were tested via Log-rank test.

**Results:**

WAZ, HAZ, and WHZ distributions remained below WHO reference curves throughout 6–60 months. Mean WAZ declined by 0.75 (NBW) and 0.40 (LBW); HAZ improved by 0.79 and 1.51, respectively. LBW-NBW differences in WAZ and HAZ narrowed rapidly after 3 and 2 years. Underweight prevalence rose from 18.8% (6 months) to 37.0% (36 months), stabilizing thereafter. Kaplan-Meier analysis showed 96% LBW vs. 87% NBW stunted, and 81% vs. 62% underweight by 60 months (steepest declines in first 18 months).

**Conclusion:**

Malnutrition significantly affects rural Southern India children, especially in the first three years, with LBW children and girls most at risk. LBW children demonstrated persistent acute and chronic malnutrition patterns until three years, after which the gradient between LBW and NBW reduced but never equalized, indicating the need for tailored nutritional supplementation.

## 1 Introduction

Malnutrition among children has remained a global concern, particularly in low- and middle-income countries (1). In 2024, the World Health Organization (WHO) reports that, globally, 150 million and 43 million children under five years are stunted or wasted respectively (2). Nearly all stunted children reside in Asia (51% of the global total) and Africa (43% of the global total). The Joint Child Malnutrition Estimates (JME) 2025 edition reveals that less than a quarter of countries are “on track” to halve the number of children under age 5 affected by stunting by 2030 (2).

The short and long term sequalae of malnutrition are manifold. Malnutrition directly increases the risk of morbidity and mortality due to childhood infections and thereby the out-of-pocket expenditure of the families (3–5). Surviving children face significant health conditions ranging from physiological conditions like- reduced work capacity and decreased physical fitness to chronic diseases like- hypertension, dyslipidemia, and insulin resistance in adulthood (6, 7). The reversibility of effects like behavioral abnormalities, poor mental development, and school achievement is still being debated in the literature (7).

Malnutrition poses a significant and concerning challenge in India. Among children under 5, 52.6% experienced composite anthropometric failure, encompassing stunting, wasting, and underweight, according to NFHS-5 analysis, with substantially higher rates in socioeconomically disadvantaged states compared to more developed regions (8). Data from the regular National Family Health Survey indicate that there has not been a satisfactory decline in the prevalence of undernutrition in India over the last 30 years (9). Based on the findings from NFHS-5, 32.1%, 35.5%, and 19.3% of under-5 children in India were underweight, stunted, and wasted, respectively. NFHS-5 reports severe stunting at 16.6%, severe underweight at 10.5%, and severe wasting at 7.7% among India's under-5 children nationally. Regionally, stunting exceeds 40% in Bihar (42.9%) and Uttar Pradesh (40.8%), wasting peaks in Rajasthan (25.0%) and Jharkhand (24.4%), while Telangana shows moderate rates (stunting ~28%, wasting ~19%) (9).

It is noteworthy that the economic status of the family is a direct determinant of nutrition and the country has put forward considerable efforts to combat malnutrition, for example- direct nutritional supplementation programs, conditional cash transfers to mothers, improving sanitation, and increasing women empowerment in the last three decades (10–13).

Despite these efforts, the 2020 Global Nutrition Report identifies India as one of the 88 countries that will not meet its 2025 global nutrition targets (14). The significant challenges in meeting nutrition targets were the inadequate coverage of nutrition programs and the absence of well-functioning health systems (inadequacies related to planning and monitoring infrastructure, human resources, financing, supply systems, information, and governance) (15). An effective nutrition surveillance mechanism is of utmost importance to detect undernutrition in an early stage and track the effects of interventions. The existence of such a surveillance system helps in timely decision-making (16). Unfortunately, most of the data on malnutrition are cross-sectional and fail to capture the growth trajectories. Growth among children occurs in differential phases in the first five years with an initial spurt transitioning to a steadier phase. Gender and birth weight are crucial determinants playing differing roles at various stages in growth, stunting and wasting which need to be evaluated longitudinally. In this context, we evaluated the growth patterns of a birth cohort till five years of age in the Ranga Reddy district of Telangana state of India. We compared LBW vs. NBW growth patterns and malnutrition at each age (6–60 months), while delineating sex-specific trajectories, in this rural Southern India birth cohort. These longitudinal can provide insights beyond cross-sectional surveys and inform targeted policy modifications to reduce persistent childhood malnutrition.

## 2 Methods

### 2.1 Study setting and participants

We performed this prospective longitudinal analysis using the LIFE cohort study database from 2011 to 2018 (17). The LIFE cohort study is an ongoing project initiated by a non-governmental organization – SHARE (Science Health Allied Research and Education) INDIA – in a rural to peri-urban population (*n* = 49,617) residing in 10,176 households in 40 villages located in the Medchal district, located about 40 kilometers northeast of Hyderabad, India. This area features a mix of smallholder farmers and daily wage laborers engaged in rain-fed agriculture, transitioning amid rapid urbanization with emerging industrial estates, special economic zones, and improved infrastructure. The community features clustered settlements blending farmland and urban fringes. The details of the cohort recruitment and baseline profile have been published earlier (17).

Trained healthcare workers routinely follow up this geocoded cohort in the community to assess how environmental, infectious, lifestyle, metabolic and genetic factors impact birth outcomes and early childhood health and development. As part of the current study, between 2009 and 2011, 1,227 women were recruited. (70% were not yet pregnant, and 30% were in the first trimester of pregnancy). The anthropometric measurements of the children from this cohort were obtained between the periods of July 2011 to Dec 2018. We followed-up the population-based cohort to study the anthropometric outcomes for five years. Institutional ethics committee and written informed written consent from parents were obtained.

The children were followed-up as a birth-cohort from birth to five years of age. The initial follow up occurred every 6 months up to 24 months and then yearly till 60 months. We allowed a maximum extension of two months for all visits and failure to get information within this period was considered as missing for that visit.

### 2.2 Data collection

As of February 2019, 543 children aged 6–7 years had completed follow-up out of 1,275 deliveries, including eighteen intrauterine deaths and six stillbirths. A trained nurse measured birthweight and crown-heel length immediately after birth to the nearest 10 g and 0.1 cm, respectively, using the SECA scale (UNICEF). Trained field investigators (FIs) recorded the postnatal anthropometric indices for these children using a standard protocol. Measurements included weight and length/height (Now onward, we will use ‘height' for length or height) or height. The FIs measured height and weight using a portable stadiometer (SECA model 213) and a portable calibrated weighing scale (SECA model 813) to the nearest 0.1 cm and 0.1 kg, respectively. Field investigators obtained the measurements in the community and entered data through tablets in a custom designed application utilizing visual basic and “My Structured Query Language” (MySQL). The data was pre-validated during data entry. The outcome variables were stunting (Age-standardized height-for-age [HAZ < −2 standard deviation (SD), wasting (Age-standardized weight-for-height (WHZ) < −2SD], and undernutrition [Age-standardized weight-for-age (WAZ) < −2SD]. We defined all the outcome variables as per the World Health Organization (WHO) classification. Details of the LIFE Study methodology have been reported previously (17).

### 2.3 Statistical analysis

We calculated the anthropometric parameters, including WAZ, WHZ, and HAZ in WHO (World Health Organization) Anthro Software. We analyzed the data using STATA version 14.0. We summarized anthropometric indices using descriptive statistics and 95% confidence intervals (CIs). Two group differences were analyzed using a proportion test and an unpaired *t*-test between LBW and NBW for the primary objective and between boys and girls for the secondary objective after normality checking. Kaplan-Meier survival analysis was conducted to show the pattern of anthropometric indices (stunting, underweight, wasting) over time using a time-to-first-event approach, with children censored at their last follow-up visit. The difference in survival curves between the normal and low birth weight babies was tested by the Log Rank test. A *p*-value < 0.05 was considered as significant for all the statistical tests.

## 3 Results

We included data of 543 new-born children (47.7% girls, *n* = 259); 117 (21.6%) had low birth weight. The average duration of pregnancies was 38.6 weeks (SD 2.4 weeks).

### 3.1 Anthropometric indices

#### 3.1.1 Overall

**Figure 1:**
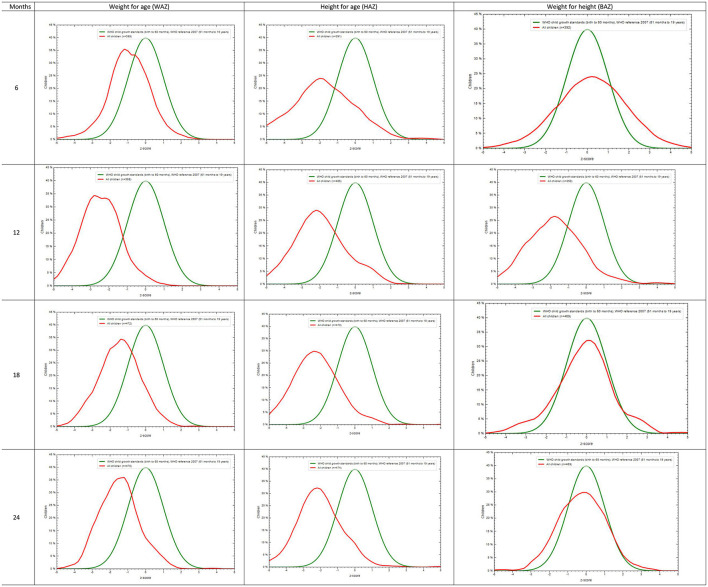
Distribution of WAZ, HAZ, and WHZ till five years of age in reference to WHO reference curve, LIFE cohort, Telangana, India. Distribution of weight-for-age Z-score (WAZ), height-for-age Z-score (HAZ), and BMI-for-age Z-score (WHZ) from birth to five years of age in the LIFE cohort (Telangana, India), plotted against WHO child growth standards.

The distributions of all three z-scores—weight-for-age (WAZ), height-for-age (HAZ), and weight-for-height (WHZ)—remained below the WHO reference curves throughout the follow-up period (Figure 1).

Mean WAZ declined from −0.98 ± 1.28 at 6 months to −1.66 ± 0.92 at 60 months; WHZ from 0.27 ± 1.91 to −1.57 ± 1.27; HAZ improved from −1.96 ± 2.02 to −1.02 ± 1.14 (Table 1).

**Table 1:** Distribution of anthropometric indices, LIFE birth cohort, Telangana, India.

z-score	Child age (months)	Overall	Birth Weight			Gender	
		Mean ±SD	Normal	Low	MD (95% CI)	Boys	Girls	MD (95% CI)
			Mean ±SD	Mean ±SD		Mean ±SD	Mean ±SD	
Weight for age (WAZ)	6	−0.98 ± 1.28	−0.82 ± 1.22	−1.58 ± 1.32	0.77 (0.47, 1.07)^*^	−0.62 ± 1.26	−1.34 ± 1.18	0.72 (0.48, 0.96)^*^
	12	−1.27 ± 1.23	−1.14 ± 1.19	−1.77 ± 1.27	0.62 (0.33, 0.92)^*^	−1.06 ± 1.21	−1.50 ± 1.21	0.43 (0.19, 0.67)^*^
	18	−1.46 ± 1.18	−1.32 ± 1.15	−1.96 ± 1.16	0.63 (0.37, 0.89)^*^	−1.29 ± 1.14	−1.65 ± 1.19	0.36 (0.14, 0.57)^*^
	24	−1.47 ± 1.12	−1.48 ± 1.11	−1.43 ± 1.18	−1.48 (−0.31, 0.20)	−1.56 ± 1.16	−1.36 ± 1.08	−0.20(−0.40, 0.01)
	36	−1.65 ± 1.04	−1.52 ± 1.02	−2.13 ± 0.98	0.61 (0.39, 0.84)^*^	−1.59 ± 1.08	−1.72 ± 1.00	0.13 (−0.06, 0.32)
	48	−1.70 ± 0.95	−1.60 ± 0.97	−2.02 ± 0.80	0.42 (0.21, 0.63)^*^	−1.79 ± 0.93	−1.60 ± 0.97	−0.19 (−0.36, −0.02)^*^
	60	−1.66 ± 0.92	−1.57 ±0.90	−1.98 ± 0.92	0.41 (0.21, 0.61)^*^	−1.73 ±0.98	−1.59 ± 0.85	−0.15 (−0.31, 0.02)
Height for age (HAZ)	6	−1.96 ± 2.02	−1.68 ± 1.93	−2.99 ± 2.02	1.30 (0.84, 1.77)^*^	−1.69 ± 1.77	−2.23 ± 2.22	0.54 (0.14, 0.93)^*^
	12	−1.98 ± 1.50	−1.78 ± 1.41	−2.70 ± 1.62	0.91 (0.56, 1.26)^*^	−1.78 ± 1.47	−2.18 ± 1.50	0.40 (0.11, 0.69)^*^
	18	−2.26 ± 1.43	−2.09 ± 1.39	−2.91 ± 1.42	0.82 (0.51, 1.13)^*^	−2.16 ± 1.31	−2.38 ± 1.55	0.23 (−0.03, 0.49)
	24	−2.03 ± 1.33	−2.04 ± 1.27	−2.01 ± 1.57	−0.03 (−0.33,0.27)	−2.05 ± 1.31	−2.01 ± 1.36	−0.04 (−0.28,0.20)
	36	−1.91 ± 1.34	−1.76 ± 1.33	−2.45 ± 1.23	0.70 (0.40, 0.99)^*^	−1.94 ± 1.26	−1.87 ± 1.43	−0.06 (−0.31, 0.18)
	48	−1.45 ± 1.22	−1.32 ± 1.24	−1.93 ± 1.02	0.61 (0.35, 0.88)^*^	−1.54 ± 1.21	−1.35 ± 1.22	−0.18 (−0.41, 0.04)
	60	−1.02 ± 1.14	−0.89 ± 1.12	−1.48 ±1.10	0.59 (0.35, 0.83)^*^	−1.09 ± 1.17	−0.95 ±1.11	−0.14 (−0.34, 0.06)
Weight for height (WHZ)	6	0.27 ± 1.91	0.27 ± 1.91	0.29 ± 1.94	−0.02 (−0.49, 0.44)	0.52 ± 1.57	0.02 ± 2.19	0.50 (0.13, 0.88)^*^
	12	−0.14 ± 1.48	−0.12 ± 1.45	−0.23 ± 1.60	0.10 (−0.25, 0.46)	−0.02± 1.55	−0.28 ± 1.39	0.26 (−0.03, 0.55)
	18	−0.09 ± 1.48	−0.06 ± 1.48	−0.21 ± 1.50	0.15 (−0.18, 0.48)	0.05 ± 1.55	−0.24 ± 1.40	0.28 (−0.01, 0.55)^*^
	24	−0.26 ± 1.29	−0.27 ± 1.29	−0.21 ± 1.30	−0.7 (−0.36, 0.22)	−0.40 ± 1.33	−0.11 ± 1.22	−0.29 (−0.52, −0.05)^*^
	36	−0.60 ± 1.41	−0.56 ± 1.40	−0.76 ± 1.46	0.20 (−0.11, 0.51)	−0.49 ± 1.32	−0.73 ± 1.50	0.25 (−0.01, 0.50)
	48	−1.14 ± 1.29	−1.14 ±1.32	−1.16 ± 1.19	0.02 (−0.26, 0.31)	−1.19 ±1.33	−1.08 ± 1.25	−0.11 (−0.35, 0.13)
	60	−1.57 ± 1.27	−1.56 ± 1.29	−1.61 ± 1.22	0.05 (−0.23, 0.32)	−1.64 ± 1.34	−1.50 ± 1.20	−0.14 (−0.36, 0.09)

^*^Statistically significant; MD, Mean Difference.

#### 3.1.2 NBW vs. LBW

Unpaired t-tests showed NBW children had significantly higher mean WAZ than LBW at most time points (MD 0.41–0.77, all *p* < 0.05), except 24 months (Table 1). Proportion tests showed underweight prevalence significantly higher in LBW at 6–18 and 36–60 months (PD 17.4–24.2, all *p* < 0.05) (Table 2). Stunting remained significantly higher in LBW except 24 months (PD 18.6–32.8, all *p* < 0.05) (Table 2). HAZ was significantly higher in NBW across all ages (MD 0.59–1.30, all *p* < 0.05) (Table 1).

**Table 2:** Distribution of the undernutrition based on birth weight and gender, LIFE birth cohort, Telangana, India.

Indicators	Child age (months)	Overall	Birth Weight			Gender		
		n (%)	Normal	Low	PD (95% CI)	Boys	Girls	PD (95% CI)
			n (%)	n (%)		n (%)	n (%)	
Underweight	6	398 (18.8)	315 (14.3)	83 (36.1)	21.9 (10.8, 32.9)^*^	202 (10.4)	196 (27.6)	−17.2 (−24.7, −9.6)^*^
	12	401 (28.4)	318 (24.5)	83 (43.4)	18.8 (7.2, 30.5)^*^	209 (20.1)	192 (37.5)	−17.4 (−26.1, −8.7)^*^
	18	469 (33.3)	370 (29.2)	99 (48.5)	19.2 (8.4, 30.2)^*^	247 (25.9)	222 (41.4)	−15.5 (−24.0, −7.1)^*^
	24	466 (32.6)	370 (33.2)	96 (30.2)	−3.0 (−1.3, 7.3)	242 (35.1)	224 (29.9)	5.2 (−3.3,13.7)
	36	468 (37.0)	368 (31.8)	100 (56.0)	24.2 (13.4, 35.0)^*^	242 (35.0)	222 (39.2)	−4.2 (−13.0,4.5)
	48	464 (31.7)	362 (28.5)	102 (43.1)	14.7 (4.0, 25.4)^*^	246 (34.6)	218 (28.4)	6.1 (−2.3,14.5)
	60	486 (31.7)	380 (27.9)	106 (45.3)	17.4 (6.9, 27.9)^*^	252 (31.0)	234 (32.5)	−1.5 (−9.8,6.8)
Stunting	6	401 (47.1)	316 (40.2)	85 (72.9)	32.8 (21.9, 43.6)^*^	203 (39.9)	198 (54.5)	−14.6 (−24.3, −5.0)^*^
	12	405 (49.1)	321 (44.6)	84 (66.7)	22.1 (10.7, 33.6)^*^	210 (41.9)	195 (56.9)	−15.0 (−24.7, −5.4)^*^
	18	469 (58.4)	370 (54.1)	99 (74.8)	20.7 (10.7, 30.6)^*^	247 (55.1)	222 (62.2)	−7.1 (−16.0,1.8)
	24	470 (54.5)	374 (53.2)	96 (59.4)	6.2 (−4.9, 17.2)	244 (52.9)	226 (56.2)	−3.3 (−12.3,5.7)
	36	468 (44.7)	368 (40.0)	100 (62.0)	22.1 (11.3, 32.8)^*^	246 (46.3)	222 (42.8)	3.5 (−5.5,12.6)
	48	464 (33.0)	363 (28.4)	101 (49.5)	21.1 (10.3, 31.9)^*^	246 (36.2)	218 (29.4)	6.8 (−1.7,15.3)
	60	490 (16.5)	384 (12.5)	106 (31.1)	18.6 (9.2, 28.0)^*^	253 (17.8)	237 (15.2)	2.6 (−4.0,9.2)
Wasting	6	397 (9.6)	314 (9.6)	83 (9.6)	0.0 (−7.2, 7.0)	202 (3.0)	195 (16.4)	−13.4 (−19.1, −7.8)^*^
	12	401 (10.2)	318 (9.8)	83 (12.1)	2.3 (−5.4, 10.0)	209 (7.7)	192 (13.0)	−5.3 (−11.3,0.6)
	18	469 (9.2)	370 (9.2)	99 (9.1)	0.0 (−6.5, 6.3)	247 (9.3)	222 (9.0)	0.3 (−4.9,5.5)
	24	466 (8.4)	370 (8.4)	96 (8.3)	0.0 (−6.3–6.2)	242 (11.1)	224 (5.4)	5.8 (0.8,10.7)^*^
	36	468 (14.7)	368 (12.8)	100 (22.0)	9.2 (0.4, 18.0)^*^	246 (11.4)	222 (18.5)	−7.1 (−13.6, −0.6)^*^
	48	462 (22.9)	361 (22.4)	101 (24.8)	2.3 (−7.1, 11.8)	245 (27.3)	217 (18.0)	9.4 (1.8, 16.9)^*^
	60	486 (32.7)	380 (32.1)	106 (34.9)	2.8 (−7.4, 13.0)	252 (33.7)	234 (31.6)	2.1 (−6.2,10.4)

^*^Statistically significant; PD, Proportion Difference.

#### 3.1.3 Boys vs. girls

Boys had significantly higher WAZ, HAZ, and WHZ at 6 months by unpaired *t*-tests (MD 0.50–0.72, all *p* < 0.05) (Table 1); WAZ/WHZ differences narrowed with girls surpassing (non-significant) by 60 months, while HAZ differences attenuated but favored boys. Underweight was significantly lower in boys at 6–18 months by proportion tests (PD −15.5 to −17.2, all *p* < 0.05) (Table 2). Stunting was significantly lower in boys at 6–12 months (PD −14.6 to −15.0, *p* < 0.05) (Table 2). Wasting was lower in boys at 6 months (PD −13.4, *p* < 0.05) but higher by 24–48 months (Table 2).

### 3.2 Survival analysis

**Figure 2:**
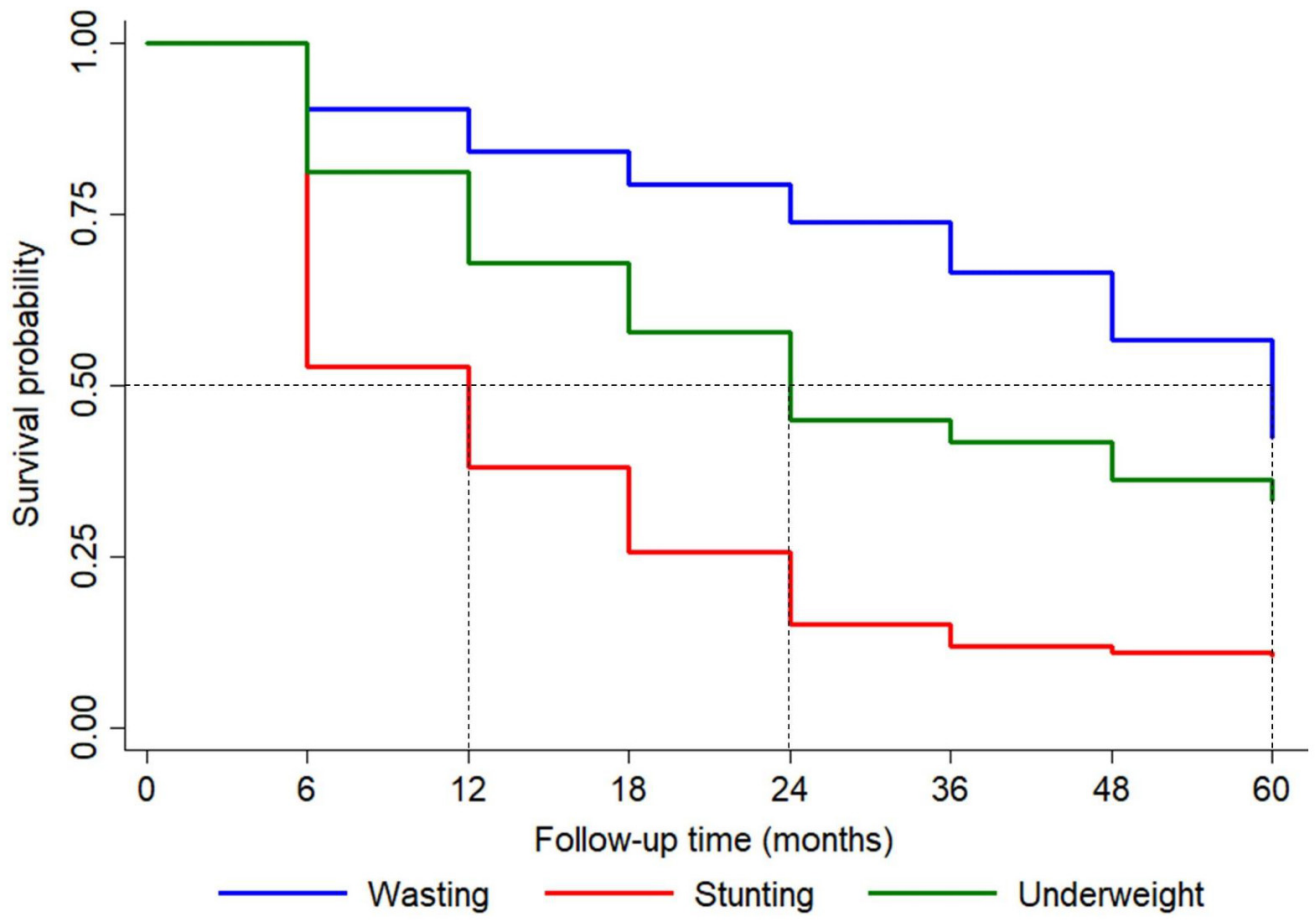
Kaplan-Meier survival curve for different undernutrition types, LIFE cohort, Telanagana, India. Kaplan-Meier estimates of survival (time to event) for different undernutrition types in the LIFE cohort (Telangana, India).

From six months to five years of age, the proportion of children who developed stunting at least one time was 89.3% (95% CI: 85.5%, 92.6%). The proportion was 66.6% (95% CI: 61.3%, 71.8%) and 57.6% (95% CI: 51.8%, 63.5%) for underweight and wasting. The probability of stunting reached to 84.9% by two years of age. At the same time, the probability of underweight and wasting reached to 55.1% and 26.2% (Figure 2).

The risk was more for girls and LBW-children for all three anthropometric indices (Tables 3, 4).

**Table 3:** Survival function and Hazard ratio (HR) for anthropometric indices in respect to gender, LIFE birth cohort, Telangana, India.

Variable	Months	Boys			Girls			Log rank test	Hazard ratio
z-score		Event	Censored	SF (95%CI)	Event	Censored	SF (95%CI)		
Underweight	6	21	0	0.90 (0.85, 0.93)	54	0	0.72 (0.66, 0.78)	0.001	1.55 (1.20–2.00)
	12	23	15	0.78 (0.72, 0.83)	30	11	0.57 (0.50, 0.64)		
	18	19	10	0.68 (0.61, 0.74)	17	5	0.48 (0.40, 0.54)		
	24	28	14	0.51 (0.44, 0.58)	15	12	0.39 (0.31, 0.46)		
	36	4	4	0.48 (0.41, 0.55)	5	5	0.35 (0.28, 0.42)		
	48	9	8	0.42 (0.34, 0.49)	5	5	0.31 (0.24, 0.38)		
	60	2	45	0.40 (0.32, 0.47)	4	28	0.27 (0.20, 0.34)		
Stunting	6	81	0	0.60 (0.53, 0.66)	108	0	0.45 (0.38, 0.52)	0.03	1.27 (1.02–1.57)
	12	27	9	0.47 (0.40, 0.53)	32	9	0.29 (0.23, 0.36)		
	18	28	2	0.32 (0.25, 0.38)	16	4	0.20 (0.14, 0.26)		
	24	23	7	0.19 (0.13, 0.25)	12	4	0.12 (0.07, 0.17)		
	36	8	2	0.13 (0.08, 0.18)	0	0			
	48	0	4	0.13 (0.08, 0.18)	2	0	0.10 (0.06, 0.15)		
	60	1	11	0.12 (0.07, 0.17)	0	11	0.10 (0.06, 0.15)		
Wasting	6	6	0	0.97 (0.94, 0.99)	32	0	0.84 (0.78, 0.88)	0.012	1.46 (1.09–1.96)
	12	12	16	0.91 (0.86, 0.94)	13	15	0.77 (0.70, 0.82)		
	18	10	11	0.86 (0.80, 0.90)	7	7	0.73 (0.66, 0.79)		
	24	12	21	0.79 (0.72, 0.84)	7	18	0.69 (0.62, 0.75)		
	36	8	8	0.73 (0.66, 0.79)	13	10	0.59 (0.52, 0.66)		
	48	14	8	0.63 (0.55, 0.70)	11	7	0.50 (0.42, 0.58)		
	60	19	57	0.47 (0.38, 0.55)	14	41	0.38 (0.29, 0.46)		

**Table 4:** Survival function and Hazard ratio (HR) for anthropometric indices in respect to birth weight, LIFE birth cohort, Telangana, India.

Variable	Months	Normal Birth Weight			Low Birth Weight			Log rank test	Hazard ratio
z-score		Event	Censored	SF (95%CI)	Event	Censored	SF (95%CI)		
Weight for age	6	45	0	0.86 (0.81, 0.89)	30	0	0.64 (0.53, 0.73)	< 0.001	1.88 (1.41–2.51)
	12	40	23	0.73 (0.68, 0.78)	13	3	0.48 (0.37, 0.58)		
	18	25	12	0.64 (0.59, 0.69)	11	3	0.34 (0.24, 0.44)		
	24	39	25	0.49 (0.44, 0.55)	4	1	0.28 (0.18, 0.38)		
	36	8	9	0.46 (0.40, 0.52)	1	0	0.26 (0.17, 0.37)		
	48	9	11	0.41 (0.35, 0.47)	5	2	0.19 (0.11, 0.28)		
	60	6	63	0.38 (0.31, 0.44)	0	10	0.19 (0.11, 0.28)		
Height for age	6	127	0	0.6 (0.54, 0.65)	62	0	0.27 (0.18, 0.37)	< 0.001	1.61 (1.25–2.07)
	12	51	17	0.44 (0.38, 0.49)	8	1	0.18 (0.1, 0.26)		
	18	39	6	0.3 (0.25, 0.35)	5	0	0.11 (0.06, 0.19)		
	24	30	11	0.18 (0.14, 0.23)	5	0	0.05 (0.20, 0.11)		
	36	7	2	0.14 (0.10, 0.19)	1	0	0.04 (0.01, 0.10)		
	48	2	3	0.13 (0.09, 0.18)	0	1	0.04 (0.01, 0.10)		
	60	1	20	0.13 (0.09, 0.17)	0	2	0.04 (0.01, 0.10)		
Wasting	6	30	0	0.90 (0.87, 0.93)	8	0	0.9 (0.82, 0.95)	0.757	1.06 (0.74–1.51)
	12	20	25	0.84 (0.8, 0.88)	5	6	0.84 (0.75, 0.91)		
	18	13	13	0.80 (0.75, 0.84)	4	5	0.79 (0.68, 0.86)		
	24	15	34	0.74 (0.68, 0.79)	4	5	0.73 (0.62, 0.82)		
	36	14	15	0.68 (0.62, 0.73)	7	3	0.62 (0.50, 0.72)		
	48	20	11	0.58 (0.51, 0.64)	5	4	0.54 (0.41, 0.65)		
	60	27	77	0.43 (0.36, 0.49)	6	21	0.42 (0.29, 0.54)		

## 4 Discussion

The study prospectively looked at the pattern of malnutrition in a rural Indian cohort from birth till five years of age. While the prevalence of underweight and wasting increased from six months to five years of age, the prevalence of stunting reduced substantially during the same time. This trend was observed in those with normal-birthweight and low-birth-weight infants and among the boys and girls. We observed that the prevalence is higher among the low-birth weight children and girls in all malnutrition types at different time points. A high gap in prevalence was noticed between the girls and the boys for all types of malnutrition during the initial age; however, the gap reduced with increase in age. Notably, every nine out of ten children in this populations suffered with stunting in this populations; 95% of them developed it within two years of age.

The proportion of underweight children in our study is comparable to the findings of others who reported that nearly one-third to two-fifths of children in India are underweight. (9, 10, 18) The increase in the proportion of underweight children over the first three years of life indicates nutritional deprivation during this critical period of growth. LBW demonstrate a pattern of chronic malnutrition until three years of age, following which the gradient between NBW and LBW reduces but never catches up, underscoring persistent catch-up deficits and indicating the need for a tailored approach to nutritional supplementation in early years and in LBW. LBW infants can benefit from targeted interventions such as fortified complementary feeding and micronutrient supplementation to mitigate long-term stunting and cognitive impairments. The documentation of this trend in both normal- and low-birth weight infants highlights the need to closely monitor growth in the first three years of life for all children, especially those with low birth weight. However, prevention of LBW remains the paramount priority, necessitating upstream interventions like improved maternal nutrition during preconception and pregnancy, enhanced antenatal care, and poverty alleviation to address fetal origins of poor growth. While broad enhancements to existing schemes, such as India's Integrated Child Development Services (ICDS) and POSHAN Abhiyaan, the substantive recommendation for LBW-specific supplementation is justified by its greater efficacy in promoting growth recovery compared to universal approaches, without diverting from preventive priorities.

On the contrary to undernutrition and wasting, which increased in proportion with time, the prevalence of stunting reduced between 36 months and 60 months, with the proportion falling from two-fifths to about a tenth. This rate remained relatively stable up to 18 months, then declined steadily up to 60 months; nearly one-third of those with low birthweight had stunting at 60 months of age. This shows a clear difference in the proportion of children with stunting between those with a normal vs. low birthweight. Strikingly, the height-for-age analysis in the survival curve showed that nearly nine out of ten children experienced stunting in the low-birth-weight group which is an alarming situation. For wasting, the cross-sectional prevalence increased abruptly after 18 months of age for normal and low birth weight babies. However, the change in the incidence of wasting was not out of proportion. Contrary to our finding, evidence from Asian settings indicated that the proportion of children with low birth weight is at significantly more risk of wasting compared to normal birth weight babies (19–21). Given our findings that underweight and stunting burden is significantly higher among low birth weight, there is a need to explore, identify and target the factors contributing to both acute and chronic malnutrition.

Malnutrition in the early stages of life, especially within the first 1,000 days, has significant implications (22). Major growth and neurodevelopment take place at this time. If failed to take place, the growth and development may be permanently damaged, is directly related to increasing childhood morbidity and mortality, increases the risk of chronic diseases in adulthood, and reduces life-long productivity (22–24). Therefore, it is critical to identify, prevent and treat any form of malnourishment at the early stage of life.

National surveys like the National Family Health Survey (NFHS-5), Comprehensive National Nutrition Survey (CNNS), and National Nutrition Monitoring Bureau (NNMB) document India's malnutrition burden. Growth chart monitoring is incorporated in the “mother and child card” to screen and refer children with Malnutrition (25). However, the regularity of this system is questionable as the training and supervision of the village-level health workers often require considerable improvement. Nutritional programs are being implemented, but despite these, malnutrition prevails (26, 27). Nutritional surveys lack longitudinal depth to dissect LBW-specific faltering in rural Southern contexts. Our study fills this by prospectively quantifying persistent deficits and sex-disparate trajectories through 60 months, offering evidence for precision nutrition programs beyond aggregate monitoring. Point-in-time prevalence reveals substantially higher stunting, wasting, and underweight rates in this rural Southern India cohort compared to national averages from national surveys, particularly among LBW children. This underscores the need for region- and birthweight-targeted interventions beyond cross-sectional survey data. Malnutrition despite national programs filling gaps indicates other major social and health system determinants must be considered to combat it. Evidence from a similar setting suggests that nutritional supplementation can be integrated with other public health programs to have favorable outcomes (13).

### 4.1 Strength and limitations

This study provides a critical message on how the growth occurs during the first five years of life. This study also provides the longest prospective comparison (6–60 months) of LBW vs. NBW growth patterns and malnutrition trajectories in rural Southern India, using point-wise tests and survival curves to delineate sex-specific data, filling critical gaps in regionally granular, longitudinal evidence from this high-burden area. Nevertheless, our study had a few limitations. We had considerable attrition from the original pregnancy cohort that might influence the outcome. At 24 months follow-up, we noticed a dip in the outcomes of the normal birth-weight babies, but we could not explain the phenomenon biologically. We also could not exclude a chance of measurement bias during the examination of the children. However, we ensured that the same data collectors collected the information according to the prescribed research protocol. All analyses are unadjusted and do not account for confounders; thus, interpretations are limited to crude associations, with adjusted models planned for future investigations.

## 5 Conclusion

Malnutrition in under-five children is a significant problem in rural settings in south India. The condition affects children within the first three years of life,-with a higher burden among girls, and the low-birth-weight children.Close monitoring of growth is required during this period to detect malnutrition early and initiate remedial measures with the potential to offset detrimental consequences of undetected and untreated malnutrition.

## Data Availability

The datasets generated for this study are available in the Supplementary material.

## References

[1] Kambale RM Francisca IN. Optimising the management of acute malnutrition. Lancet Glob Health. (2022) 10:e453–4. doi: 10.1016/S2214-109X(22)00087-0 35303443

[2] Nutrition and Food Safety. Available online at: https://www.who.int/teams/nutrition-and-food-safety/monitoring-nutritional-status-and-food-safety-and-events/joint-child-malnutrition-estimates/latest-estimates (Accessed April 08, 2026).

[3] Njuguna RG Berkley JA Jemutai J. Cost and cost-effectiveness analysis of treatment for child undernutrition in low- and middle-income countries: a systematic review. Wellcome Open Res. (2020) 5:62. doi: 10.12688/wellcomeopenres.15781.1 33102783 PMC7569484

[4] Siddiqui F Salam RA Lassi ZS Das JK. The intertwined relationship between malnutrition and poverty. Front Public Health. (2020) 8:453. doi: 10.3389/fpubh.2020.00453 32984245 PMC7485412

[5] Tahsina T Ali NB Hoque DME Huda TM Salam SS Hasan MM . Out-of-pocket expenditure for seeking health care for sick children younger than 5 years of age in Bangladesh: findings from cross-sectional surveys, 2009 and 2012. J Health Popul Nutr. (2017) 36:33. doi: 10.1186/s41043-017-0110-4 28893323 PMC5594455

[6] Zhang YY Chen BX Wan Q. Global, regional, and national burden of nutritional deficiencies spanning from 1990 to 2021, with a focus on the impacts observed during the COVID-19 pandemic. Front Nutr. (2025) 12:1535566. doi: 10.3389/fnut.2025.1535566 40406157 PMC12094969

[7] Martins VJB Toledo Florêncio TMM Grillo LP do Carmo P Franco M Martins PA Clemente APG . Long-lasting effects of undernutrition. Int J Environ Res Public Health. (2011) 8:1817–46. doi: 10.3390/ijerph8061817 21776204 PMC3137999

[8] Shah G Siddiqa M Shankar P Karibayeva I Zubair A Shah B. Decoding India's child malnutrition puzzle: a multivariable analysis using a composite index. Children. (2024) 11:902. doi: 10.3390/children11080902 39201837 PMC11352507

[9] Ministry of Health and Family Welfare. India Fact Sheet National Family Health Survey- 5. Available online at: http://rchiips.org/nfhs/NFHS-5_FCTS/India.pdf (Accessed March 23, 2026).

[10] Murarkar S Gothankar J Doke P Pore P Lalwani S Dhumale G . Prevalence and determinants of undernutrition among under-five children residing in urban slums and rural area, Maharashtra, India: a community-based cross-sectional study. BMC Public Health. (2020) 20:1559. doi: 10.1186/s12889-020-09642-0 33066763 PMC7565769

[11] Narayan J John D Ramadas N. Malnutrition in India: status and government initiatives. J Public Health Policy. (2019) 40:126–41. doi: 10.1057/s41271-018-0149-5 30353132

[12] Kapil U Chaturvedi S Nayar D. National nutrition supplementation programmes. Indian Pediatr. (1992) 29:1601–13. 1291517

[13] Kinra S Rameshwar Sarma KV. Ghafoorunissa null, Mendu VVR, Ravikumar R, Mohan V, et al. Effect of integration of supplemental nutrition with public health programmes in pregnancy and early childhood on cardiovascular risk in rural Indian adolescents: long term follow-up of Hyderabad nutrition trial. BMJ. (2008) 337:a605. doi: 10.1136/bmj.a605 18658189 PMC2500199

[14] Global Nutrition Report. Global Nutrition Report. Available online at: https://globalnutritionreport.org/reports/2020-global-nutrition-report/ (Accessed March 23, 2026).

[15] Paul VK Sachdev HS Mavalankar D Ramachandran P Sankar MJ Bhandari N . Reproductive health, and child health and nutrition in India: meeting the challenge. Lancet. (2011) 377:332–49. doi: 10.1016/S0140-6736(10)61492-4 21227494 PMC3341742

[16] Gillespie S Haddad L Mannar V Menon P Nisbett N. The politics of reducing malnutrition: building commitment and accelerating progress. Lancet. (2013) 382:552–69. doi: 10.1016/S0140-6736(13)60842-9 23746781

[17] Kusneniwar G Whelan RM Betha K Robertson JM Ramidi PR Balasubramanian K . Cohort Profile: the longitudinal indian family health (LIFE) pilot study, Telangana State, India. Int J Epidemiol. (2017) 46:788–789j. doi: 10.1093/ije/dyw174 27649805 PMC5837391

[18] Prusty RK Bairwa M Anwar F Mishra VK Patel KK Mangal DK. Socio-biomedical predictors of child nutrition in India: an ecological analysis from a nationally representative demographic and health survey, 2015–2016. J Health Popul Nutr. (2022) 41:1. doi: 10.1186/s41043-021-00273-8 34980283 PMC8722359

[19] Rahman MS Howlader T Masud MS Rahman ML. Association of low-birth weight with malnutrition in children under five years in Bangladesh: do mother's education, socio-economic status, and birth interval matter? PLoS ONE. (2016) 11:e0157814. doi: 10.1371/journal.pone.0157814 27355682 PMC4927179

[20] Abbas F Kumar R Mahmood T Somrongthong R. Impact of children born with low birth weight on stunting and wasting in Sindh province of Pakistan: a propensity score matching approach. Sci Rep. (2021) 11:19932. doi: 10.1038/s41598-021-98924-7 34620917 PMC8497567

[21] Huey SL Finkelstein JL Venkatramanan S Udipi SA Ghugre P Thakker V . Prevalence and correlates of undernutrition in young children living in urban slums of Mumbai, India: a cross sectional study. Front Public Health. (2019) 7:191. doi: 10.3389/fpubh.2019.00191 31355176 PMC6639755

[22] Scott JA. The first 1000 days: a critical period of nutritional opportunity and vulnerability. Nutr Diet. (2020) 77:295–7. doi: 10.1111/1747-0080.12617 32478460

[23] Horta BL Victora CG Organization WH. Short-Term Effects of Breastfeeding: a Systematic Review on the Benefits of Breastfeeding on Diarrhoea and Pneumonia Mortality (2013). Available online at: https://apps.who.int/iris/handle/10665/95585 (Accessed March 23, 2026).

[24] Vyas S. A systematic review on nutritional vulnerability and opportunity during the first 1000 days of life for ensuring better human capital. Indian J Sci Technol. (2021) 14:2511–6. doi: 10.17485/IJST/v14i30.1253

[25] Ramachandran P Gopalan HS. Assessment of nutritional status in Indian preschool children using WHO 2006 Growth Standards. Indian J Med Res. (2011) 134:47–53. 21808134 PMC3171917

[26] Anganwadi Feeding Programme by Akshaya Patra. Available online at: https://www.akshayapatra.org/anganwadi-feeding (Accessed March 23, 2026).

[27] Anaemia Mukt Bharat. Government of India (2022). Available online at: https://pib.gov.in/PressReleasePage.aspx?PRID=1795421 (Accessed March 23, 2026).

